# A genosensor for detection of HTLV-I based on photoluminescence quenching of fluorescent carbon dots in presence of iron magnetic nanoparticle-capped Au

**DOI:** 10.1038/s41598-018-32756-w

**Published:** 2018-10-22

**Authors:** Mohadeseh Zarei-Ghobadi, Sayed-Hamidreza Mozhgani, Fariba Dashtestani, Amir Yadegari, Fatemeh Hakimian, Mehdi Norouzi, Hedayatollah Ghourchian

**Affiliations:** 1 Institute of Biochemistry and Biophysics, University of Tehran, Tehran, Iran; 2 Department of Virology, School of Public Health, Tehran University of Medical Sciences, Tehran, Iran; 3 Department of Microbiology, School of Medicine, Alborz University of Medical Sciences, Karaj, Iran; 4 Research Center for Clinical Virology, Tehran University of Medical Sciences, Tehran, Iran; 5 Department of Developmental Sciences, Marquette University School of Dentistry, Milwaukee, WI 53233 USA

## Abstract

Carbon dots and Fe_3_O_4_@Au were synthesized to develop a new biosensor to detect DNA target. We investigated the photoluminescence property of carbon dots (CDs) in the presence of Fe_3_O_4_-capped Au (Fe_3_O_4_@Au). Firstly, we designed two dedicated probes for unique long sequence region of human T-lymphotropic virus type 1 genome. One of the probes was covalently bound to the CDs. In the absence of target, CDs-probe was adsorbed on the surface of Fe_3_O_4_@Au through two possible mechanisms, leading to quenching the fluorescence emission of CDs. The fluorescence emission of CDs was recovered in the presence of target since double-stranded DNA cannot adsorb on the Fe_3_O_4_@Au. Also, Fe_3_O_4_@Au can adsorb the unhybridized oligonucleotides and improves the accuracy of detection. The specificity of the proposed biosensor was confirmed by BLAST search and assessed by exposing the biosensor to other virus targets. The experimental detection limit of the biosensor was below 10 nM with linear range from 10 to 320 nM.

## Introduction

Nanomaterial-based detection methods have been developed to open novel and simple routes toward improving point-of-care diagnoses^1,2^. One of the main challenges to reach this achievement is utilizing simple methods and environmentally-friendly materials. Carbon nanostructures are known as beneficial biomaterials due to their optical and electrochemical characterizations^3^. Moreover, it has been reported that functionalization of carbon nanostructures with biomolecules can significantly ameliorate their performance. Therefore, carbon nanostructures have extensively employed in various biomedical applications including biosensors, drug and gene delivery systems, bioimaging, and tissue scaffold reinforcement^3,4^.

In the recent decades, biosensors have attracted numerous attentions in pathogen diagnosis, disease progression, point-of-care monitoring of treatment, and drug discovery^5^. Different techniques accompany with using signal amplification labels have been used to develop the selective and sensitive biosensors. Among them, biosensors relying on the fluorescence emission measurement and hybridization between target DNA and labelled probe oligonucleotides facilitate rapid and sensitive detection of biomolecules. Although, organic fluorophores have good features of photostability and high efficiency, they suffer from high cost and photobleaching effect^6,7^. One of the proper substitution to the traditional fluorophores is quantum dot nanoparticles (QDs) due to their unique traits, like high quantum yields and photostability, broad excitation, narrow emission, and excellent resonance energy transfer^8,9^. However, the application of QDs confronts with problems such as intricate and costly synthesis steps, and toxicity^10^. Thus, fluorescent carbon dots have attracted researchers due to their photo- and chemical stability, low toxicity and cost, and biocompatibility^11^.

The other useful and applicable nanoparticles in biomedical research are magnetic nanoparticles (MNPs), which usually coated by polymers or other metals to increase their stability in various physiological pHs^12^. However, their magnetization should be conserved after surface modification. The coating of MNPs with gold shell causes the surface stabilization, biocompatibility, and magnetic property preservation^13^.

According to the advantage of inimitable properties of carbon dots (CDs) and also their fluorescence quenching in the proximity of quenchers, several biosensors have designed for oligonucleotides detection. Bai *et al*. presented a biosensor utilizing methylene blue (MB) as a quencher of CDs through adsorption on the surface of CDs^14^. With addition of DNA, the intensity of CDs fluorescence were restored, since MB bound DNA and removed it from the CDs. Huang *et al*. developed a radiometric nanosensor based on the quenching fluorescence of CDs in the presence of ethidium bromide (EB)^15^. Upon the addition of DNA, the fluorescence of EB was significantly increased but the fluorescence intensity of CDs remained constant. Qadarre *et al*. introduced a HIV-1 gene sensor based on higher association tendency of the CDs-labeled oligonucleotides to the target rather than AuNPs/graphene oxide nanocomposite, which caused the recovery of the quenched fluorescence of CDs^16^. However, the mentioned biosensors either used organic compounds that were not specific for the oligonucleotide sequences or designed for detection of single-stranded oligonucleotide target.

Human T-lymphotropic virus type 1 (HTLV-1) is only known retrovirus, which can cause cancer in human and develop two diseases including adult T-cell leukemia/lymphoma (ATLL) and HTLV-1-associatedmyelopathy/tropical spastic paraparesis (HAM/TSP)^17^. The detection of HTLV-1 can be performed by polymerase chain reaction (PCR), serological methods, and western blot^18^. The early detection of HTLV-1 is momentous, as it can escape from the host defense mechanisms. The aforementioned methods need sample preparation, high cost and tied with the false positive results^19–21^. To best of our knowledge, the biosensor based on quenching the fluorescence emission of CDs in proximity of iron magnetic nanoparticles capped-Au and its different affinity to single-stranded DNA and double-stranded DNA has not been reported.

In this study, we develop a simple method for the synthesis of carbon dots (denoted as CDs). Furthermore, we survey the fluorescence property of the prepared CDs and their fluorescence quenching in presence of the synthesized nanoparticles coated by a gold layer (denoted as Fe_3_O_4_@Au). Afterwards, we present an inexpensive, versatile, and sensitive method for detection of oligonucleotides that is part of a special region of HTLV-1. To this end, we designed two specific probes to diagnosis target DNA. One of the probes was functionalized with CDs. In the absence of target, CDs-probe was adsorbed on the surface of Fe_3_O_4_@Au, resulted in quenching the fluorescence emission of CDs which was retrieved in the presence of target.

## Results

### Principle of Sensing

**Figure 1:**
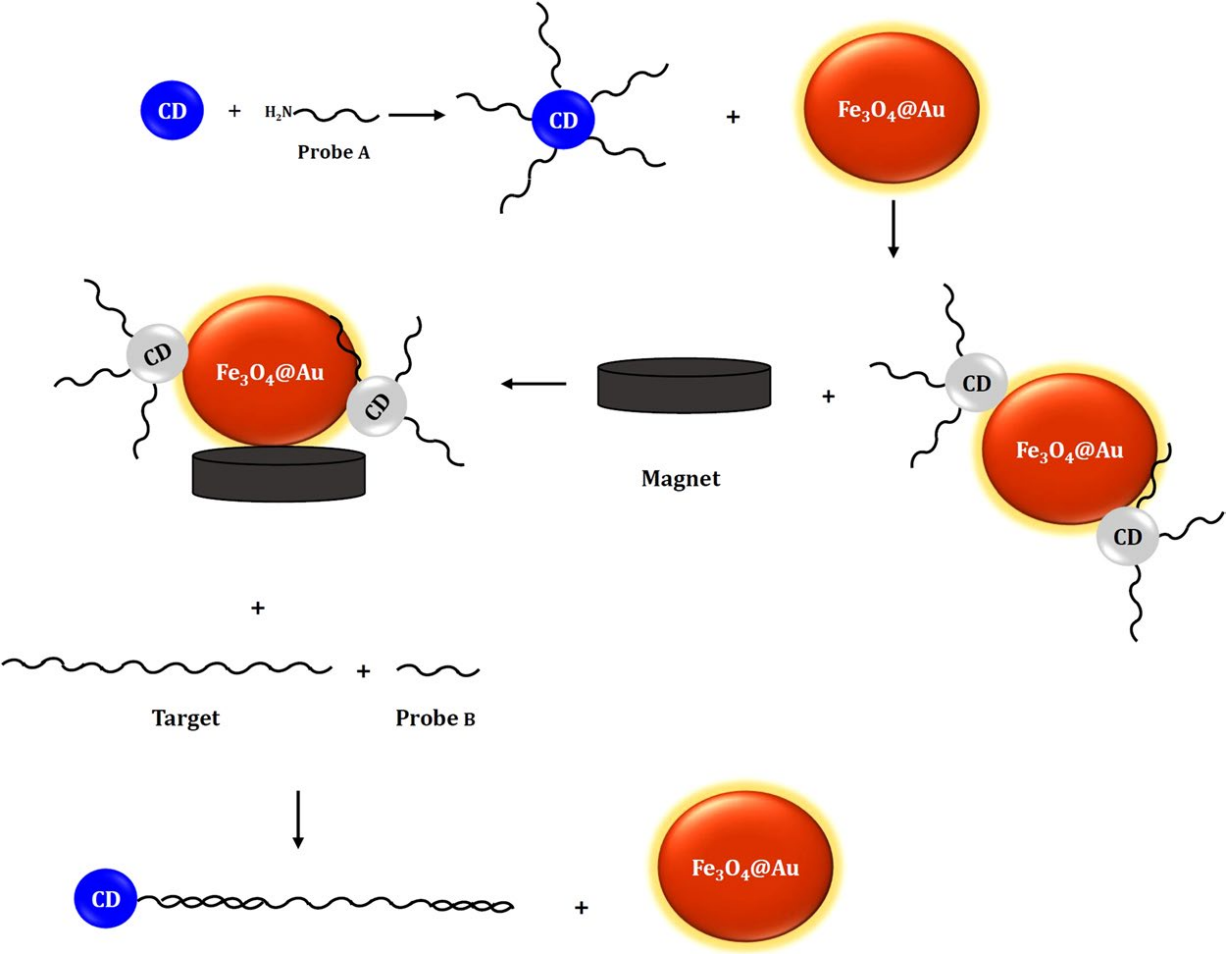
Schematic illustration of the detection steps of DNA target.

Figure 1 displays the sensing principal of the proposed biosensor. Firstly, the synthesized CDs are functionalized with the aminated probe A. The addition of Fe_3_O_4_@Au leads to two possible interactions, including the electrostatic adsorption of CDs-probe A through negative charge of CDs or adsorption of probe nucleotides^22^. As a result, the interaction between CDs-probe A and Fe_3_O_4_@Au leads to fluorescence quenching of CDs. In the presence of target and probe B, the hybridization occurs and double-stranded DNA (dsDNA) containing CDs-probe A, target, and probe B constitutes. Finally, the unhybridized probes and targets that adsorbed on the Fe_3_O_4_@Au were separated using a magnet. Therefore, the fluorescence emission of C-dots was recovered, since dsDNA does not adsorbed on the surface of Fe_3_O_4_@Au.

### Characterization of Fe_3_O_4_@Au and CDs

**Figure 2:**
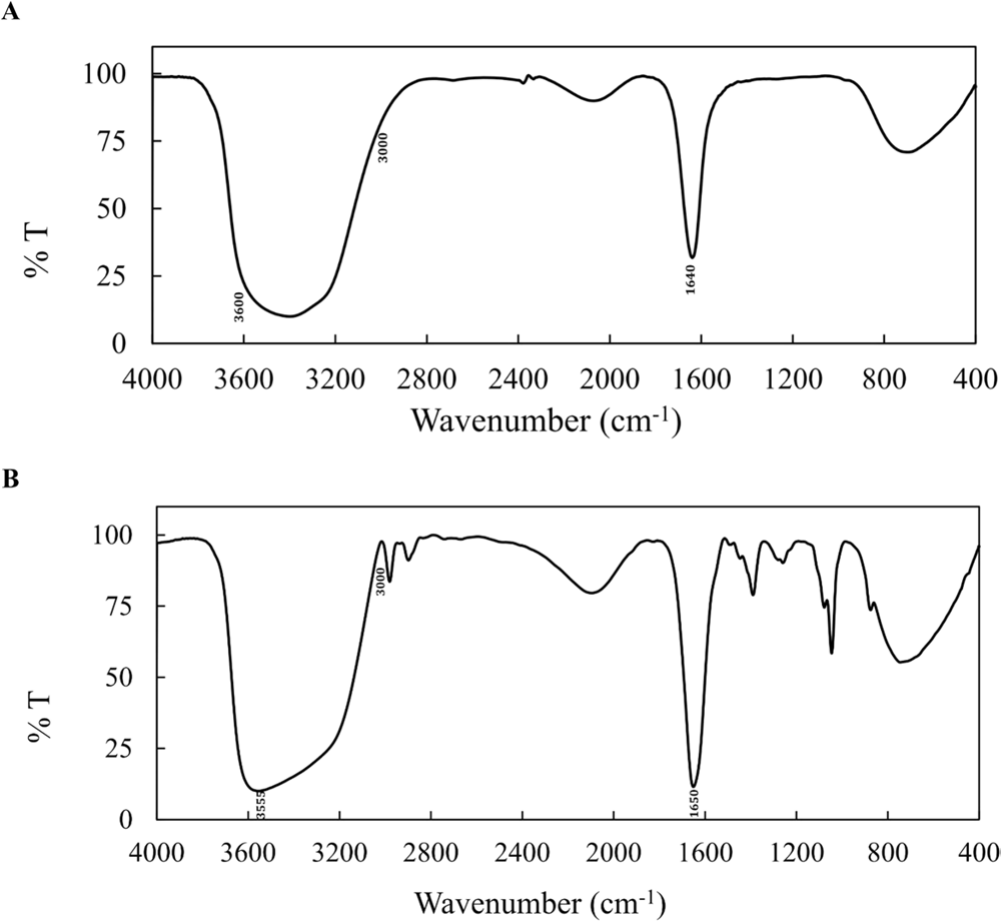
The FT-IR spectra of (**A**) Fe_3_O_4_@Au and (**B**) CDs.

FT-IR spectrometry was employed to identify types of functionality of ligands attached to the nanoparticles. Figure 2A displays the FT-IR spectrum of Fe_3_O_4_@Au. The presence of carboxylic group on Fe_3_O_4_@Au is confirmed according to the distinctive band at 1640 cm^−1^ while the band from 3000 to 3500 cm^−1^ can be assigned to O–H stretching vibration. The FT-IR spectrum of CDs is shown in Fig. 2B. The carbonyl stretching frequency of carboxylic functional group on CDs is at 1650 cm^−1^. Also, the O–H stretching vibration band is around 3000 to 3600 cm^−1^. The stretch vibrational band at 3555 cm^−1^ is related to the N–H, which is overlapped with O–H stretching vibration and become wider. Hence, the FT-IR spectrum of CDs confirms the presence of carboxyl and amine functional groups present on the surface of CDs.

**Figure 3:**
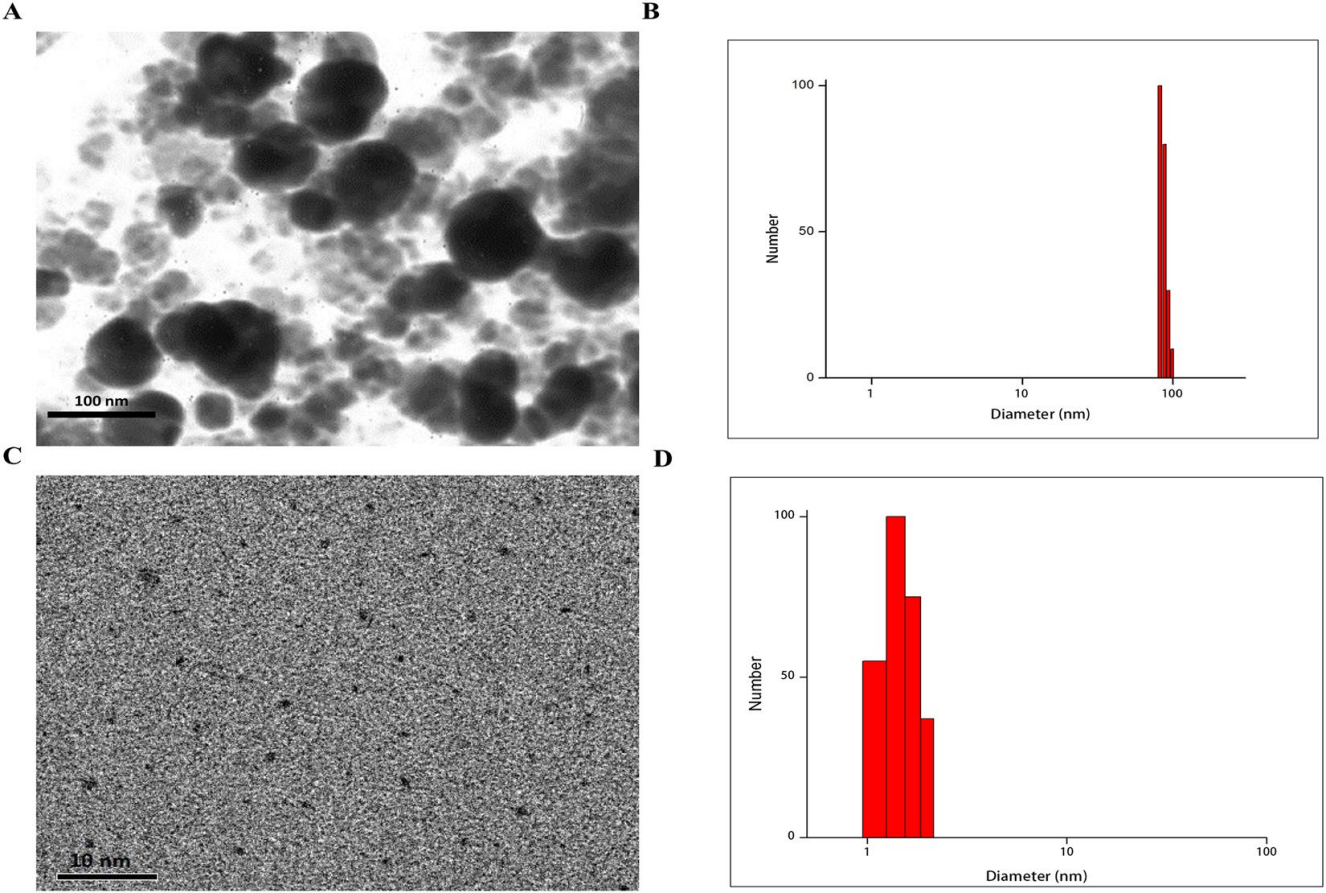
(**A**)TEM image and (**B**) DLS analysis of Fe_3_O_4_@Au; (**C**) TEM image and (**D**) DLS analysis of CDs.

TEM image and DLS analysis showed that Fe_3_O_4_@Au were of spherical shape and around 80–90 nm in dimeter (Fig. 3A,B). Also, the TEM image and DLS analysis of the synthesized CDs revealed that the mean size of the synthesized nanoparticles is to be about 1.5 nm with a good monodispersity without any noticeable agglomeration suggesting successful formation of CDs (Fig. 3C,D). The zeta potential values of Fe_3_O_4_@Au and CDs were found to be +3 ± 0.5 mV and −11 ± 0.5 mV, respectively. The negative charge of CDs can be related to the presence of carboxyl groups.

**Figure 4:**
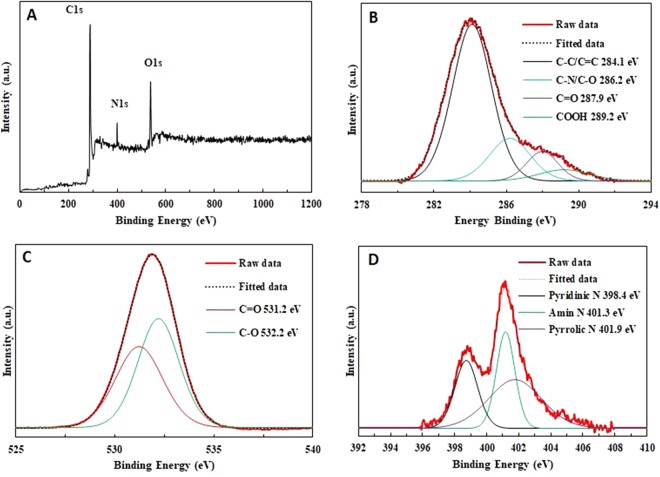
(**A**) XPS survey spectra of CDs, (**B**) C_1s_ high-resolution XPS spectra, (**C**) O_1s_ high resolution XPS spectra and (**D**) N_1s_ high-resolution XPS spectra of CDs.

X-ray photoelectron spectroscopy (XPS) was used for element and surface composition analyses of carbon dots. The full spectra (Fig. 4A) indicates three typical major peaks at 284, 400, and 530 eV corresponding to C1s, N1s, and O1s, respectively^23,24^. In addition, the C_1s_ spectrum presented in Fig. 4B, shows four peaks at 284.1, 286.2, 287.9, and 289.2 eV which are ascribed to C-C/C=C, C–N/C–O, carbonyl carbons (C=O), and carboxyl carbons (COOH), respectively (23). The O_1s_ band as shown in Fig. 4C, demonstrates two peaks at 531.2 and at 532.3 eV for C=O and C−O, respectively. Figure 4D reveals the deconvolution of N_1s_ into three peaks indicating pyridinic N (298.4 eV), amino N (401.3 eV), and pyrrolic N (401.9 eV).

### The quenching of CDs fluorescence in the proximity of Fe_3_O_4_@Au

**Figure 5:**
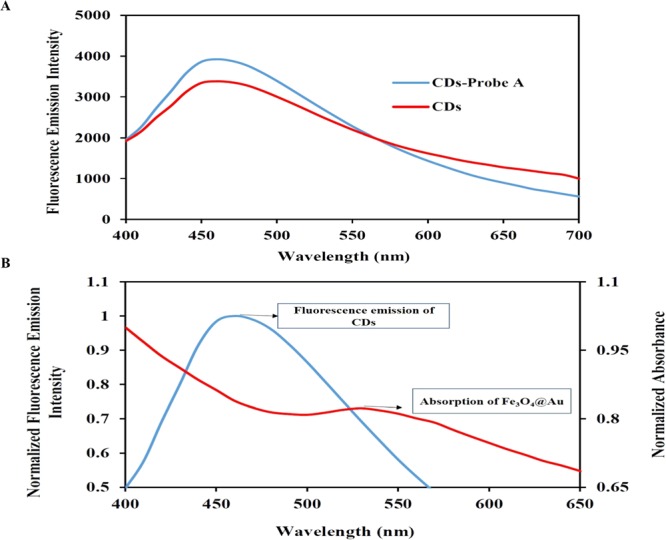
(**A**) The fluorescence emission spectra of CDs before and after conjugation with probe A. (**B**) The fluorescence emission spectra of CDs and UV-Vis absorption spectra of Fe_3_O_4_@Au solution.

**Figure 6:**
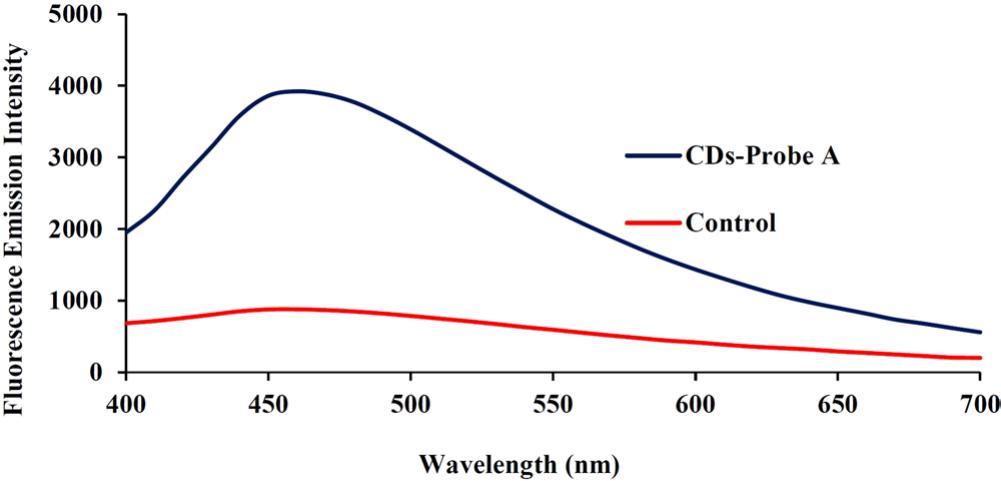
The fluorescence emission spectra of CDs-probe A before (control) and after adsorption on the Fe_3_O_4_@Au surface.

The emission intensity of CDs was measured under 380 nm excitation before and after conjugation with probe A. To measure the fluorescence emission, the peak intensity in the range of 400–700 nm was followed. Figure 5A shows that the emission peak of CDs was increased slightly after conjugation with oligonucleotides of probe A. The overlapping between fluorescence emission spectra of CDs and absorption spectra of Fe_3_O_4_@Au confirms the fluorescence quenching of CDs (Fig. 5B). The pristine CDs-probe A showed a strong fluorescence emission spectrum at about 460 nm with excitation in 380 nm, while it was quenched after adsorption on the Fe_3_O_4_@Au (Fig. 6). The proposed biosensor met the required conditions for the Forster resonance energy transfer (FRET) mechanism which includes: (i) overlapping the emission spectra of the fluorophore (energy donor) with the absorption of the quencher (energy acceptor); (ii) the required closeness of donor and acceptor (<10 nm); iii) dipole-dipole interaction. The FRET efficiency was calculated as 0.8 magnitude according the equation QE = 1 − (F_DA_/F_D_), where F_DA_ denotes the integrated fluorescence intensity of the donor in the presence of the acceptor and F_D_ shows the integrated fluorescence intensity of the donor alone^25^.

### Construction of biosensor for detection of target virus gene

**Figure 7:**
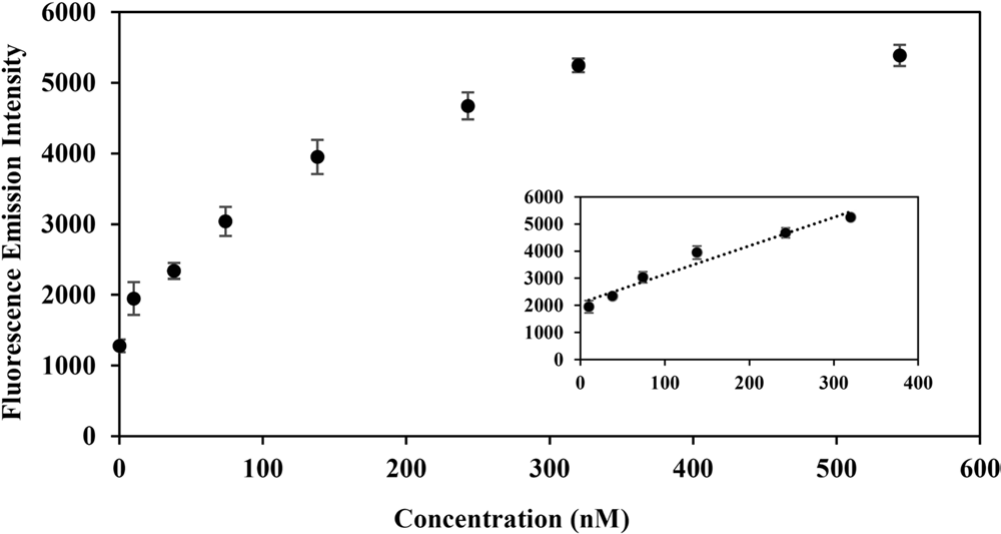
The fluorescence spectra of the CDs-probe A as a function of target concentrations. The inset shows the linear range of the biosensor response.

The efficiency of the biosensor for target HTLV-1 gene detection was surveyed. To this end, different concentrations of target from 0 to 544 nM were prepared and analyzed according to the mentioned principle of sensing. As shown in Fig. 7, the fluorescence emission intensity of CDs retrieves by increasing the target concentration. The linear range was to be determined from 10 to 320 nM with a limit of detection equals to 10 nM (inset of Fig. 7).

### Specificity of the biosensor

**Figure 8:**
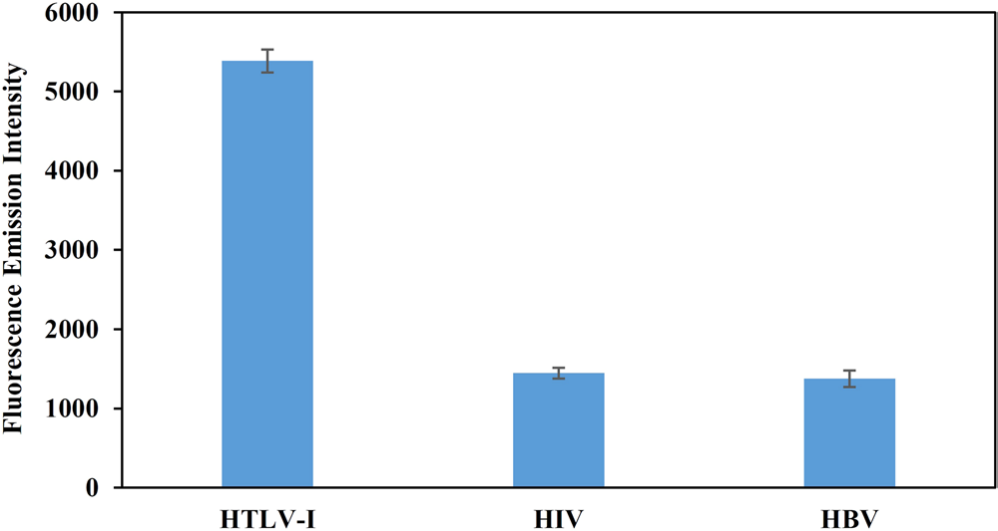
Specificity of the biosensor for human T-lymphotropic virus-1 (HTLV-1) rather than human immunodeficiency virus (HIV) and hepatitis B virus (HBV). The concentration of all samples was 544 nM.

To explore the specificity of the biosensor toward HTLV1 target, the genes of hepatitis B virus (HBV) and human immunodeficiency virus (HIV) were also considered as the target. However, the probes were designed for a specific region of HTLV1 gene and their alignment with the complete genome of the virus revealed its specificity for HTLV1. Nevertheless, Fig. 8 confirms that fluorescence emission recovery of CNs-probe A decreases significantly in the presence of other viruses. Actually, the hybridization of probes with non-complementary targets does not occur, resulted in non-recovery of fluorescence emission of CDs.

## Discussion

In this study, a novel and cost-effective genosensor was introduced based on fluorescence quenching of CDs in the proximity of Fe_3_O_4_@Au. The sensing signal was upon changing the fluorescence emission of CDs-probe. In the absence of target, CDs-probe were adsorbed on the Fe_3_O_4_@Au surface, which caused their fluorescence quenching. However, the fluorescence emission of CDs were recovered in the presence of the target which is because of desorption from the surface of Fe_3_O_4_@Au. The major novelty of our proposed biosensor is first applying Fe_3_O_4_@Au with high surface area as an efficient quencher of CDs. Also, it was employed to adsorption of excessive probes and second strand of dsDNA target, which leads to decreasing unwanted signals to improve the accuracy of detection. In fact, in our proposed biosensor setup, the presence of complementary sequence may interfere with oligonucleotide probes which can decrease the detectable signals. Also, the Fe_3_O_4_@Au nanoparticles have some advantages that make them good candidate in biosensor applications such as ease-synthesis, high biocompatibility, cost-effectivity, large surface area, and strong adsorption ability^26^. Moreover, selecting the specific region of genome as the target sequences is important in development of a biosensor for detection of partial pathogen genome sequence. The long specific sequences can increase the efficiency of detection, Herein, we chose the 122-base fragment of the tax region of the HTLV-1 genome. Also, we designed proper complementary probes to detect it. In addition, we used the nucleotide BLAST tool (https://blast.ncbi.nlm.nih.gov/Blast.cgi) to ensure about the specificity of designed probes toward target.

In addition to the common methods of PCR and ELISA, only a few biosensors have been designed for HTLV-1 DNA detection including based on electrochemical (LOD = 1.71 pM, 11.3 aM)^27,28^ and fluorescence (LOD = 8.5 nM, 19.5 pg/μl)^18,29^ techniques. However, these biosensors need complex sample preparations and also multiple steps to final detection. The present simple method can be modified in future studies to detect wide concentration ranges of other genomic biomolecules.

## Methods

### Synthesis of Fe_3_O_4_ nanoparticles and Fe_3_O_4_@Au

Fe_3_O_4_ nanoparticles were synthesized by co-precipitation of ferric and ferrous salts^30,31^. In brief, 0.1 g FeCl_3_ g and 0.04 g FeCl_2_ were dissolved into 20 mL of deoxygenated deionized water under N_2_ gas. After stirring for 10 minutes at 50 °C, 5 mL of NaOH solution (0.3 M) were added gradually while vigorously stirring until its color changed from orange to black. Then, the mixture was stirred for an additional 1 hour and gradually cooled down to room temperature. After separation of the black product with a permanent magnet, the precipitate was washed 3 times with 70 mL of deionized water. To avoid Fe_3_O_4_ aggregation, 0.02 g of tetraethylammonium perchlorate was added to the synthesized nanoparticles. For synthesis of Fe_3_O_4_@Au, 4 mL of HAuCl_4_ (14 mM) was added into prepared Fe_3_O_4_ nanoparticles solution and then heated to boiling under stirring. Then, 6 mL of sodium citrate (%1) was added into the reaction mixture. The mixture was boiled under stirring until the color of solution changed from black to burgundy. The reaction mixture was boiled under stirring for 5 min^32^.

### Synthesis of carbon dots

Carbon dots were easily synthesized through one step hydrothermal method. Briefly, 0.5 g of o-phenylenediamine was dissolved in 20 ml of ethanol at room temperature under vigorous stirring. Then, 50 ml of deionized water was added to the mixture and poured into a 100 ml Teflon container. The reactor was subsequently put in an oven at 200 °C for 24 h. After that, the reactor was naturally cooled down to room temperature and the product was dialyzed using 500 KDa dialysis bag for 3 days. The final product was kept in 4 °C for future use.

### Oligonucleotide design

Two probes A and B, which were complementary to two specific region of HTLV-1, were designed by Gene Runner software (version 6.5.48). The nucleotide BLAST tool (https://blast.ncbi.nlm.nih.gov/Blast.cgi) was utilized to confirm the specificity of the probes. The designed probes were completely specified for the 122-base fragment of the tax region of the HTLV-1 genome. The DNA oligonucleotides (Probe A: 5′-CAGCCATCTTTAGTACTACAGTCCTCCTCC-(T)10-NH_2_-3′) and Probe B: 5′-TTCCGTTCCACTCAACCCTCAC-3′ were purchased from Takapouzist Biotech Company (Iran). DNA target sequences was as follow:

5′-GGAGGAGGACTGTAGTACTAAAGATGGCTGGCCATCTTTAGGGCAGGGCCCGGAAATCAT

AGGCGTGCTATCGGTAAATGTCCAAATAAGGCCTGGAGTGGTGAGGGTTGAGTGGAACGGAA-3′

### Preparation of CDs-oligonucleotides conjugation

The probe A was functionalized with CDs according to the following procedure: The as-synthesized CDs solution was sonicated for 15 min. Then, 2 µL EDC (400 µg/mL) and 2 µL NHS (320 µg/mL) were added and incubated for 1 hr. After that, 50 µL probe A was added and again incubated for 2 hr.

### Characterization

UV-Vis absorption spectra were recorded using a Varian Cary Bio 100 spectrophotometer. Fourier transforms infrared (FTIR) spectral analyses of nanoparticle in KBr disc were recorded using a Perkin-Elmer 343 spectrometer (USA). The images of nanoparticles were recorded by transmission electron microscopy electron microscope (TEM, Philips, EM 208). XPS analysis was performed using a hemispherical analyser supplied by an Al Ka X-ray source (operating at energy of 1486.6 eV in a vacuum higher than 10_7 Pa) and the deconvolution of signals was done by Gaussian components. Dynamic light scattering (DLS, 90 plus Brookhaven Instruments Corporation, USA) was used for size and charge determinations. Zeta potential measurements were attained by a ZetaPALS analyzer (Brookhaven Instruments).

#### Fluorescence measurement

The fluorescence measurements were performed on a fluorescence microplate reader (H4, Bio Tech Co, USA) at room temperature. The excitation was set at 380 nm and the emission spectra was recorded from 400 to 700 nm with both excitation and emission slits of 5 nm.

The blank in the absence of target was considered as Fe_3_O_4_@Au and deionized water and in the presence of target as deionized water and then subtracted from the corresponding sample to correct the fluorescence background.

### The sensing procedure of target

In order to target detection, 20 µL CDs-probe A and 20 µL Fe_3_O_4_@Au were mixed and incubated for about 30 min at room temperature. After separation with magnet, the supernatant was removed. Then, the fluorescence emission of CDs in equivalent volume of deionized water was measured. Afterwards, 20 µL probe B and different concentrations of target were added. The Fe_3_O_4_@Au was collected by magnet and upper supernatant was extracted to further fluorescence emission measurement.

## Data Availability

The datasets generated during and/or analysed during the current study are available from the corresponding author on reasonable request.
